# Insulin-like Growth Factor I Couples Metabolism with Circadian Activity through Hypothalamic Orexin Neurons

**DOI:** 10.3390/ijms23094679

**Published:** 2022-04-23

**Authors:** Jaime Pignatelli, M. Estrella Fernandez de Sevilla, Jacob Sperber, Daniel Horrillo, Gema Medina-Gomez, Ignacio Torres Aleman

**Affiliations:** 1 Cajal Institute-CSIC, 28002 Madrid, Spain; mefernandezdesevilla@cajal.csic.es (M.E.F.d.S.); jspers9@gmail.com (J.S.); 2 CIBERNED, 28029 Madrid, Spain; 3 Medicine Faculty, Autonoma University, 28049 Madrid, Spain; 4 Department of Basic Sciences of Health, Area of Biochemistry and Molecular Biology, LAFEMEX Lab, University Rey Juan Carlos, 28933 Madrid, Spain; danielhorrillo@gmail.com (D.H.); gema.medina@urjc.es (G.M.-G.); 5 Achucarro Basque Neuroscience Center, 48940 Leioa, Spain; 6 Ikerbasque Foundation for Science, 48009 Bilbao, Spain

**Keywords:** orexin, IGF-I, central control of metabolism, circadian activity, feeding entrainment

## Abstract

Uncoupling of metabolism and circadian activity is associated with an increased risk of a wide spectrum of pathologies. Recently, insulin and the closely related insulin-like growth factor I (IGF-I) were shown to entrain feeding patterns with circadian rhythms. Both hormones act centrally to modulate peripheral glucose metabolism; however, whereas central targets of insulin actions are intensely scrutinized, those mediating the actions of IGF-I remain less defined. We recently showed that IGF-I targets orexin neurons in the lateral hypothalamus, and now we evaluated whether IGF-I modulates orexin neurons to align circadian rhythms with metabolism. Mice with disrupted IGF-IR activity in orexin neurons (Firoc mice) showed sexually dimorphic alterations in daily glucose rhythms and feeding activity patterns which preceded the appearance of metabolic disturbances. Thus, Firoc males developed hyperglycemia and glucose intolerance, while females developed obesity. Since IGF-I directly modulates orexin levels and hepatic expression of KLF genes involved in circadian and metabolic entrainment in an orexin-dependent manner, it seems that IGF-I entrains metabolism and circadian rhythms by modulating the activity of orexin neurons.

## 1. Introduction

In mammals, sleep–wake and food intake cycles are tightly regulated by circadian and metabolic rhythms [1,2,3]. Circadian rhythms are driven by the hypothalamic suprachiasmatic nucleus (SCN), which is considered the pace-maker region, and by other hypothalamic and brainstem regions [3,4,5]. Metabolic rhythms are driven by clock genes from secondary brain regions, mainly located in the hypothalamus, and peripheral organs, such as the liver [3,6]. Circadian rhythms are reset by environmental light that is detected by the retina and transmitted to the SCN [5], while metabolic rhythms are reset by food intake, which is termed the “food clock” [7]. Light entrains the circadian clock through the SCN, and this synchronizes clock genes from peripheral organs through the hypothalamic–pituitary–adrenal axis [8]. In turn, metabolic clocks tune circadian clocks through hormonal (ghrelin, leptin, insulin, glucocorticoids, GLP1) and nutrient (glucose, ketone bodies, non-esterified lipids) levels, and by food anticipatory behavior [9,10]. This central and peripheral crosstalk allows organisms to coordinate internal biological functions with optimal phases of the environment, such as food availability, as stated by the “circadian resonance” theory [11].

Hypothalamic orexin neurons modulate circadian rhythms, such as the sleep–wake cycle [12,13,14], and also influence clock cell activity and time-keeping of the SCN [15]. Serum insulin-like growth factor I (IGF-I) has recently been postulated to entrain circadian and feeding rhythms at still undefined central sites [16,17,18], and modulates glucose metabolism and insulin sensitivity [19,20,21] by acting at a central site [22,23]. In turn, hypothalamic orexin neurons are involved in glucose sensing [24] and metabolism [25], feeding behavior [26], and overall energy balance [27], and their impairment is linked to obesity [28]. The activity of orexin neurons, which express IGF-I receptors, is modulated by IGF-I [29] and diverse humoral signals, including glucose and insulin [30].

Based on these observations, we postulated that IGF-I acts as a peripheral signal on orexin neurons for entrainment of circadian activity with metabolism, and its disruption leads to metabolic alterations.

## 2. Results

### 2.1. IGF-I Modulates Glucose Rhythms through Orexin Neurons

**Figure 1:**
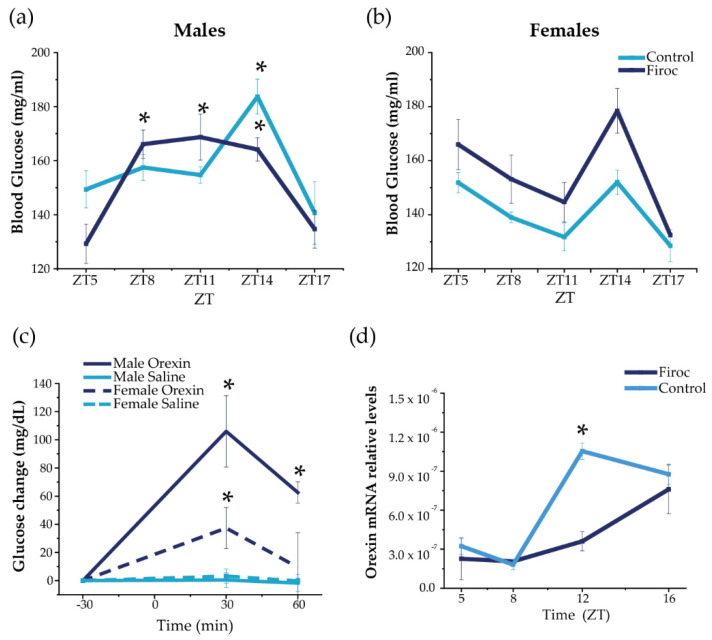
Disruption of IGF-IR in orexin neurons alters daily oscillations in blood glucose and hypothalamic orexin. Blood glucose was measured in Firoc (dark blue) mice and littermates (light blue) at different times during the 24 h cycle (ZT5, ZT8, ZT8, ZT14, and ZT16) in males (*n* = 10–15) and females (*n* = 7–10). (**a**) Male Firoc mice showed an altered glucose rhythm compared to controls (two-way ANOVA, * *p* < 0.05). In control mice, a significant peak was observed at ZT14 (two-way ANOVA and the Tukey test, * *p* < 0.05), while significantly higher levels were observed in Firoc mice from ZT8 to ZT14 (two-way ANOVA and the Tukey test, * *p* < 0.05). (**b**) Firoc females showed a glucose rhythm similar to the control. (**c**) Intracerebral (ICV) orexin (9 nmol) increases blood glucose levels in male (*n* = 5) and female (*n* = 5) mice (two-way ANOVA, * *p* < 0.05). The glucose response was significantly different in male and female mice (three-way ANOVA, * *p* < 0.05). (**d**) Orexin expression at the end of the resting phase (ZT12) was significantly higher in the control mice, but not in Firoc mice (one-way ANOVA, * *p* < 0.05); however, at ZT16, there were no differences between the two genotypes.

Daily blood glucose rhythms are centrally regulated by the circadian activity of the SCN through the autonomous nervous system and indirect efferents from the PVN and orexin neurons [6]. Indeed, orexin neurons modulate the circadian activity of SCN [15] and orexin-deficient mice lack daily glucose rhythms [30]. Circadian information from the SCN is coordinated with the circadian clocks from peripheral organs, such as the liver [31] and pancreas [32], to drive the circadian glucose rhythm. Hence, we first determined the circadian rhythm of blood glucose in Firoc mice as a readout of circadian and metabolic entrainment [6]. Serum glucose levels were measured in male and female Firoc and control mice (3–5 months old) during the light (ZT5, ZT8, and ZT11) and the dark phases (ZT14 and ZT17), under regular feeding conditions (Figure 1). Significant differences in the glucose rhythm were observed in Firoc and control males (Figure 1a; two-way ANOVA, *F*(genotype, time) = 2.63104, *p* = 0.04117). In controls, a significant peak of glucose was observed at ZT14 compared to nadir levels at ZT5 (Figure 1a; two-way ANOVA and the Tukey post-hoc test, *t*(5,14) = 3.79066, *p* = 0.01393). In Firoc mice, higher blood glucose levels were observed from ZT8 to ZT14, compared to ZT5 (Figure 1a; two-way ANOVA and the Tukey post-hoc test *t*(5,8) = 4.27201, *p* = 0.00263; *t*(5,11) = 4.57772, *p* = 8.63482 × 10^−4^; *t*(5;14) = 4.29321, *p* = 0.00243). In females, no differences were observed, although glucose levels in Firoc females were slightly, but not significantly, higher (Figure 1b; two-way ANOVA *F*(control, Firoc) = 2.983; *p* = 0.0909) and the Tukey post-hoc test *t*(genotype, ZT5) = 0.6967; *p* = 1; *t*(genotype, ZT8) = 1.1697; *p* = 1; *t*(genotype, ZT11) = 1.0665; *p* = 1); *t*(genotype, ZT14) = 1.9329; *p* = 1). 

To determine whether the observed sexual dimorphism is related to the sex-dependent regulation of glucose by orexin, we administered orexin (ICV, 3 nmol) to male and female control mice at ZT6, and measured blood glucose 30 and 60 min later, as previously described [33]. As shown in Figure 1c, ICV orexin increased blood glucose both in males (two-way ANOVA, *F*(treatment, time) = 50.32, *p* = 6.88 × 10^−8^) and females (two-way ANOVA, *F*(treatment, time) = 10.04, *p* = 0.00378). However, the response was sexually dimorphic (three-way ANOVA, *F*(sex, treatment, time) = 5.23123, *p* = 0.00821); in males, glucose levels increased for 60 min (two-way ANOVA and the Tukey post-hoc test, *t*(0,30) = −5.09811, *p* = 2.36084 × 10^−4^, *t*(0,60) = −3.68467, *p* = 0.03223), whereas glucose levels in females increased for only 30 min (two-way ANOVA and the Tukey post-hoc test, *t*(0,30) = 3.51108, *p* = 0.02381).

In turn, hypothalamic orexin shows a circadian rhythm of expression, which is low during the resting phase and high at the beginning of the active phase [34,35]. We measured hypothalamic orexin levels at ZT5 and ZT8 (during the resting phase) and at ZT12 and Z16 (during the active phase) in male mice only, as glucose alterations were more pronounced in them. We found that daily oscillations of orexin expression (Figure 1d) were altered in Firoc mice at the end of the resting phase. Orexin levels were significantly higher at ZT12 in the control mice (one-way ANOVA, *q*(WT, Firoc) = 10.05642; *p* = 5.3731 × 10^−6^), while similar levels were observed in control and Firoc mice at ZT16. These results indicate that lack of IGF-I signaling in orexin neurons of Firoc mice alters the circadian orexin rhythm, but without abrogating it.

### 2.2. IGF-I Modulates Feeding Behavior through Orexin Neurons

Orexin neurons regulate feeding behavior and energy homeostasis [36], and have been described as an efferent pathway for the food-entrainable oscillator (FEO) [37]. In turn, IGF-I seems necessary for the synchronization of circadian activity and feeding time [16] that are coordinated through the interaction of the light–dark cycle (light-entrainable oscillator; LEO) with FEO [38]. Hence, we evaluated the feeding behavior and metabolic rhythms in Firoc and control mice using metabolic cages. 

**Figure 2:**
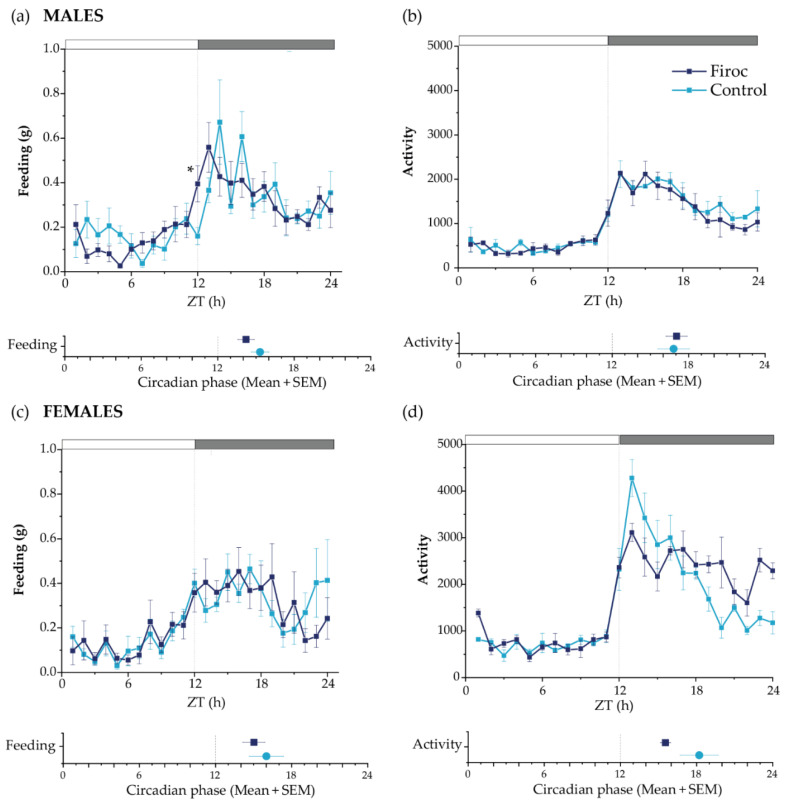
Circadian behaviors are disturbed in Firoc mice. Male (**a**,**b**) and female (**c**,**d**) Firoc (dark blue) and control (blue light) mice were phenotyped using indirect calorimetry (PhenoMaster) cages for 14 days. Food intake (**a**,**c**) and activity (**b**,**d**) were collected daily every 1 h. Data shown are the mean ± SEM for each parameter for the last 5 days at each time point. The circadian phase obtained for each parameter using the MetaCycle algorithm is shown at the bottom of each panel. Male Firoc mice showed an altered feeding behavior compared to the control mice, with an advance in feeding time (the Mann–Whitney U test, * *p* < 0.05), but no changes in their activity. Female female mice did not show significant changes in the feeding behavior or activity.

No differences in total food intake or activity patterns were seen between control and Firoc mice. However, Firoc males showed a significant difference in feeding behavior, with increased feeding at ZT12 compared to the control mice (the Mann–Whitney test, U = 5, Firoc *n* = 9; control *n* = 6; *p* = 0.00670); Figure 2a). Further, we determined the rhythmicity parameters using a bioinformatic algorithm. Firoc males showed a reduction in the feeding period, as well as a phase advance in feeding, activity, and oxygen volume consumption, together with a phase delay in RER (Figure 2a and Supplementary Table S1). Overall, it seems that IGF-I signaling is relevant for the synchronization of LEO with FEO in males. Female Firoc mice did not show differences in feeding or activity rhythms (Figure 2b,c).

**Figure 3:**
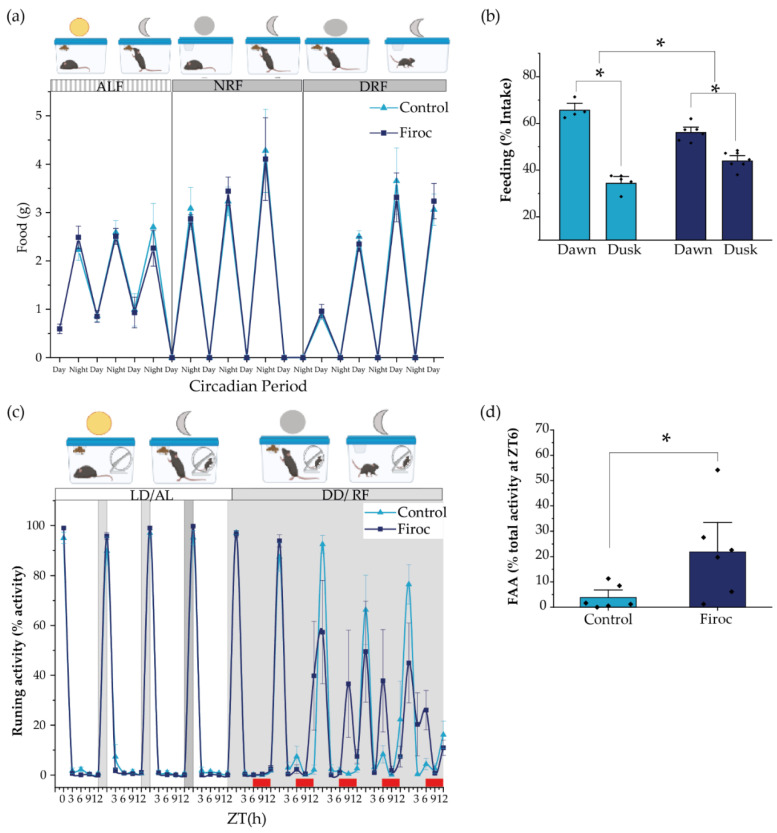
The alignment of circadian activity with the food clock is impaired in Firoc mice. (**a**) Both Firoc and control mice adapted to changes in light–food presentation. Mice were housed individually for 1 week under a regular light–dark cycle and *ad libitum* food regime (ALF). Then, the light was removed and food was restricted to the subjective night (NRF) for 4 days. After that, food availability was moved to the subjective day (DRF). Both groups spent about 4 days becoming habituated to these new conditions and started eating a similar amount of food as that consumed during the ALF experimental period. (**b**) However, analysis of the amount of food taken during the first 6 h in the morning (dawn) and the next 6 h in the afternoon (dusk) showed that control and Firoc mice mainly had meals during dawn (two-way ANOVA and the Tukey test, * *p* < 0.05), as expected, while Firoc mice showed a delayed meal (two-way ANOVA, * *p* < 0.05). (**c**) Food anticipatory activity (FAA) was measured under constant darkness after 5 days of adaptation when both control and Firoc mice ingested the same food amount. (**d**) Firoc mice showed an increased FAA 3 h before food presentation as compared to the control mice. *N* = 6–7 (two-way ANOVA, * *p* < 0.05).

Since feeding behavior is slightly but significantly altered in male Firoc mice under regular food availability and light–dark conditions, we wondered if they would show an altered FEO, which entrain energetic status with food availability. Under regular conditions, dusk and dawn meals account for the need for fuel replenishing after fasting, and to store energy substrates for the incoming resting (fasting) phase, respectively [7,39]. We placed Firoc and control mice under constant darkness to remove light entrainment, and restricted food availability for 12 h/day (from ZT0 to ZT12, during the subjective resting phase). As expected, 5 days later, Firoc and control mice synchronized their feeding behavior to the new food schedule (Figure 3a) and consumed a similar amount of food. Both control and Firoc mice had more food during dawn than during dusk (Figure 3b), as demonstrated by multi-comparison post-hoc analysis (two-way ANOVA and the Tukey post-hoc test, *t*(control, time) = 11,66, *p* < 0.0001 and two-way ANOVA, *t*(Firoc, time) = 5.55869, *p* = 0.000259). However, significant differences in the feeding time were observed between the two groups (two-way ANOVA, *F*(genotype, time) = 30.45257, *p* = 0.0000467). Firoc mice consume less food during dusk (two-way ANOVA, *t*(genotype, dusk) = −3.9029, *p* = 0.007) and more during dawn (two-way ANOVA, *t*(genotype, dawn) = 3.9029, *p* = 0.007). 

In a second experiment, we measured food anticipatory activity (FAA), a read-out of FEO, using free-running wheels as a proxy of activity. After 1 week under normal light conditions and ad libitum feeding, the light was removed, and feeding time was restricted to 6 h per day (from ZT6 to ZT12, the subjective resting phase). We measured activity at ZT0, ZT3, ZT6, ZT9, and ZT12, and FAA was calculated as the activity displayed three hours before food supply (between ZT3 and ZT6). Both control and Firoc mice adapted to the new feeding schedule within a few days, showing increased activity 3 h before food presentation (Figure 3c). However, differences between Firoc and control mice were observed (two-way ANOVA, *F*(genotype) = 5.796, *p* = 0.0215). FAA was significantly higher in Firoc mice by the fifth day (one-way ANOVA, *F*(genotype) = 6.9603, *p* = 0.023; Figure 3d), suggesting an enhanced FEO entrainment in these mice, as compared to the control mice.

### 2.3. IGF-I Modulates Metabolic Homeostasis through Orexin Neurons

**Figure 4:**
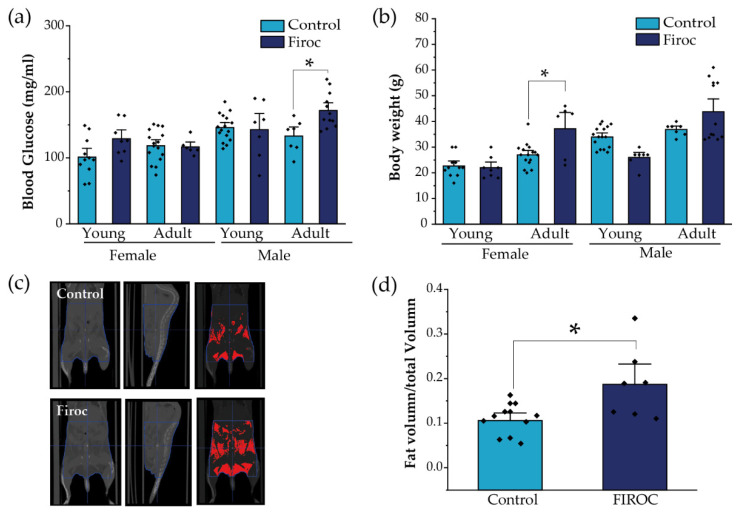
Sex-dependent metabolic disturbances in Firoc mice. (**a**) Blood glucose levels after 6 h fasting were significantly increased in adult Firoc males (two-way ANOVA, * *p* < 0.05), but not in females. (**b**) Bodyweight was significantly heavier only in female Firoc mice (two-way ANOVA, * *p* < 0.05), in an age-dependent manner. (**c**) A representative computerized tomography (CT) scan in control and Firoc females is shown. Fat depots are shown in red in the right pictures. (**d**) Fat accumulation was calculated from CT scans and shown to be significantly increased in adult Firoc females (one-way ANOVA; * *p* < 0.05).

Since the disturbed entrainment of circadian and metabolic rhythms has been associated with a greater risk to develop metabolic disorders, we evaluated whether alterations in feeding behavior combined with a loss of glucose rhythms in Firoc mice result in altered metabolic homeostasis. First, we measured basal glucose levels at ZT6, after 6 h of fasting, in young (less than 3 months) and adult (older than 6 months) mice. Again, whereas Firoc females did not show differences in blood glucose levels at any age, Firoc males gradually developed hyperglycemia with age (two-way ANOVA, *F*(genotype, age) = 5.83483, *p* = 0.02003). Adult Firoc mice showed higher glucose levels than controls (two-way ANOVA and the Tukey post-hoc test, *t*(control, adult) = 3.09907, *p* = 0.0205; Figure 4a). Conversely, Firoc females showed increased weight gain with age (two-way ANOVA *F*(genotype, age) = 4.79308, *p* = 0.03598). Adult Firoc females gained more weight than control females (two-way ANOVA and the Tukey post-hoc test, *t*(control adult, Firoc adult) = 3.6137, *p* = 0.00613; Figure 4b). No weight differences were observed between Firoc and control male mice at any age. To determine whether the accumulation of fatty tissue underlies weight gain in adult Firoc females, we analyzed fat distribution using CT scanning and found that they accumulate more visceral and epididymal fat tissue than control littermates (one-way ANOVA, *t*(control, Firoc) = −2.84825, *p* = 0.01221; Figure 4c,d). Histological preliminary analysis did not reveal hyperplasia of adipocytes (not shown) in Firoc mice with respect to the control mice. Consequently, fat accumulation seems to be a result of an increase in the number of adipocytes in female Firoc mice.

**Figure 5:**
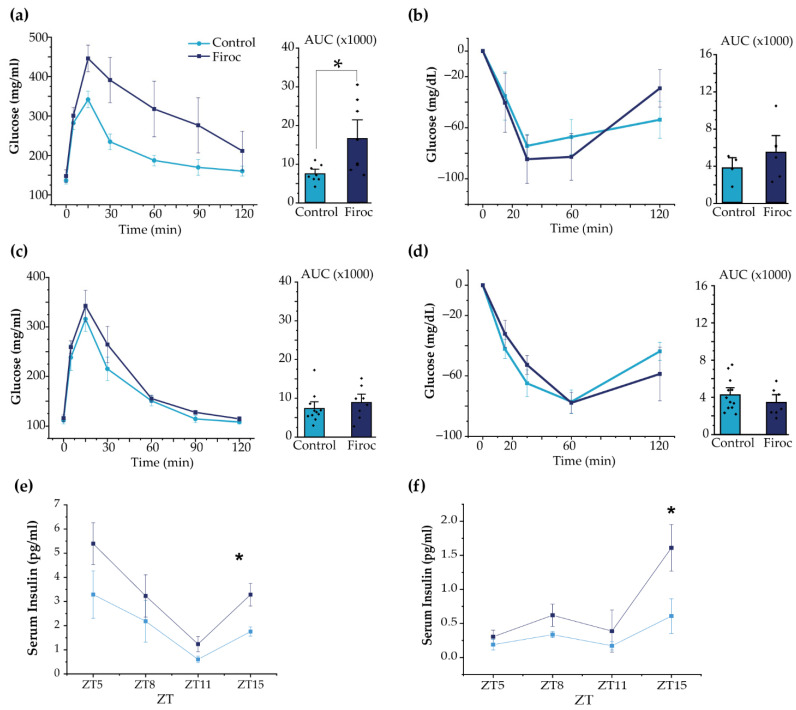
Sexual dimorphism of glucose homeostasis in Firoc mice. (**a**) Glucose tolerance test in male mice. Blood glucose levels and quantification histograms (area under the curve (AUC) are shown. (**b**) Insulin tolerance test in males. Blood glucose levels and quantification histograms (area under the curve (AUC) are shown. (**c**) Glucose tolerance test in females. Blood glucose levels and quantification histograms are shown. (**d**) Insulin tolerance test in females. Blood glucose levels and quantification histograms are shown. Firoc males (**a**), but not females (**c**), showed glucose intolerance (one-way ANOVA, * *p* < 0.05) compared to their respective controls. Glucose levels after systemic insulin administration remained within normal values in both male (**b**) and female (**d**) Firoc mice. Serum insulin levels were determined by ELISA at ZT5, ZT8, ZT11, and ZT15 in male (**e**) and female (**f**) Firoc and control mice. Both male and female Firoc mice showed significantly higher insulin levels at ZT15 (one-way ANOVA; * *p* < 0.05).

In agreement with the above results, adult (Figure 5a), but not young (Figure S1a), Firoc males displayed glucose intolerance (one-way ANOVA, *F*(genotype, AUC) = 7.44681, *p* = 0.01631). Insulin sensitivity was not changed in Firoc males, although glucose levels returned to baseline more rapidly (Figure 5b and Figure S1b). Conversely, neither adult nor young Firoc females showed altered glucose (Figure 5c and Figure S1c, respectively) or insulin sensitivity (Figure 5d and Figure S1d, respectively). 

Although glucose handling in response to insulin load was not affected in Firoc mice, we determined whether basal levels of insulin could be affected in Firoc mice, since their glucose daily rhythm was affected. We analyzed serum insulin levels at ZT5, ZT8, ZT12, and ZT16. As shown (Figure 5e,f), serum insulin levels were significantly higher at ZT15 in both male (one-way ANOVA; *F*(Firoc; control) = 9.21654; *p* = 0.01616) and female mice (one-way ANOVA; *F*(Firoc; control) = 12.43179; *p* = 0.01681), indicating that Firoc mice develop hyperinsulinemia, at least during the active phase.

To confirm the central role of IGF-I signaling on orexin neurons in the regulation of glucose homeostasis, we used a previously described recombinant AAV-HCRT-CRE virus to express CRE recombinase, specifically in orexin neurons of igfrf/f mice [40]. Mice injected with AAV-HCRT-CRE showed glucose intolerance in the glucose tolerance test (Figure S3), as compared to mice injected with an AAV control virus (one-way ANOVA, *t*(AAV-HCRT-CRE, AAV-control) = −3.56676, *p* = 0.00442). This reinforces a regulatory role of IGF-I on orexin responses to glucose increases.

### 2.4. IGF-I Modulates Hypothalamic Orexin and Liver KLF Expression

**Figure 6:**
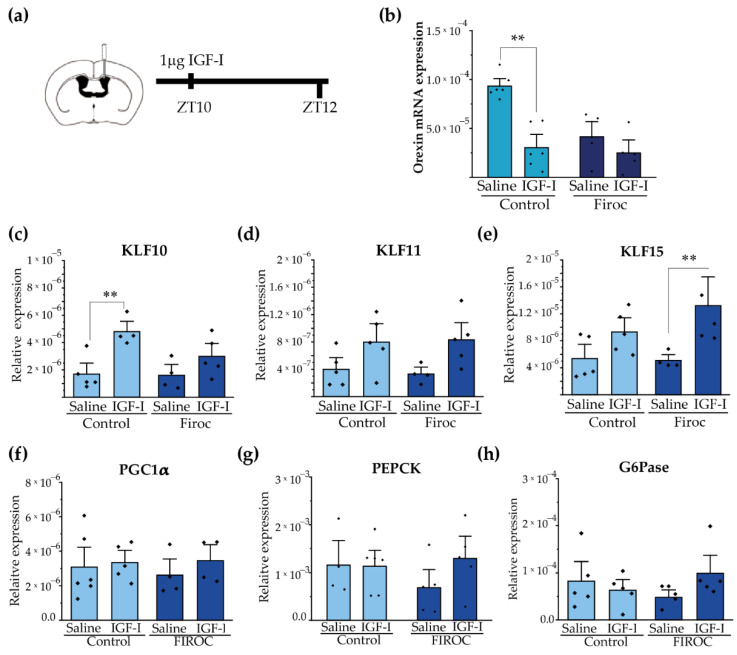
Central administration of IGF-I modulates hypothalamic orexin and liver KLF10 expression. (**a**) Intracerebroventricular (ICV) cannulas were placed in control (*n* = 10) and Firoc mice (*n* = 10). After 1 week of recovery, 1 µg of IGF-I or saline was injected ICV at ZT10 and mice were sacrificed 2 h later. Transcriptional changes in the hypothalamus (**b**) and in the liver (**c**–**h**) were analyzed by real-time PCR. In the hypothalamus (**b**), IGF-I administration downregulated orexin mRNA transcription in the control mice, but not in Firoc mice. In the liver, IGF-I significantly increased KLF-10 (**c**) expression in the control mice, but not in FIROC mice, whereas (**e**) IGF-I upregulated KLF-15 in FIROC mice, but not in the control mice (two-way ANOVA, ** *p* < 0.05).

Since Firoc mice show reduced orexin levels [29], we determined whether IGF-I regulates them. After ICV injection of IGF-I (1 µg) at ZT8, we analyzed the expression of orexin in the hypothalamus at ZT12 (Figure 6a). We observed that IGF-I blunted orexin mRNA levels in the control mice (two-way ANOVA and the Tukey post-hoc test, *t*(control saline, control IGF-I) = 5.5609, *p* = 0.000168), but not in Firoc mice (two-way ANOVA and the Tukey post-hoc test, *t*(Firoc saline, Firoc IGF-I) = 1.32427, *p* = 1). Confirming a direct effect of IGF-I onto orexin neurons, IGF-I (1 nM) decreased orexin mRNA levels in primary hypothalamic neuronal cultures (Figure S2).

Synchronization of hepatic and body circadian clocks is partially mediated by hepatic Krüppel-Jacob transcription factors (KLF), which are directly regulated by circadian clocks. We analyzed whether central IGF-I regulates the KLF transcriptional network in the liver through orexin neurons. We focus on KLF-10, KLF-11, and KLF-15 as they have been reported to regulate glucose metabolism. Intracerebroventricular IGF-I injection significantly increased liver KLF-10 (Figure 6c) transcription in the control mice (two-way ANOVA and the Tukey post-hoc test, *t*(control saline, control IGF-I) = −3.6099; *p* = 0.0475) but not in Firoc mice (two-way ANOVA and the Tukey post-hoc test, *t*(Firoc saline, Firoc IGF-I) = −1.48221; *p* =0.95393). Conversely, ICV IGF-I increased liver expression of KLF-15 (Figure 6e) in Firoc (two-way ANOVA and the Tukey post-hoc test, *t*(Firoc saline, Firoc IGF-I) −3.01123; *p* = 0.0497), but not in the control mice (two-way ANOVA and the Tukey post-hoc test, *t*(control saline, control IGF-I) = −1.71716; *p* = 0.63145). No significant differences were observed in liver KLF11 (Figure 6d) after IGF-I administration. ICV IGF-I administration did not modify the expression of gluconeogenic genes PGC1a (Figure 6f), PEPCK (Figure 6g), or G6Pase (h) in the control mice. In Firoc mice we observed tendencies of upregulation of PEPCK and G6Pase, but they did not reach significancy.

## 3. Discussion

In mammals, cellular circadian clocks are entrained with the light–dark cycle to synchronize biological processes in a tissue-specific fashion. These processes underlie different circadian rhythms, such as the sleep–wake and fasting–feeding 24 h cycles. Hence, light–dark information is coupled to energy status to synchronize activity with feeding behavior.

Orexin neurons are reciprocally connected with the circadian [15], and the food clock regulatory circuits [37] participate in the regulation of sleep–wake rhythms [12,29,41] and feeding behaviors [26]. Indeed, orexin neurons have been described as circadian [15,42] and food-entrained oscillators [37]. We previously reported that a loss of IGF-I control on orexin neurons causes abnormal sleep patterns [29] and behavioral disorders [40]. Now, we provide evidence that IGF-I tunes orexin neurons for the proper synchronization of circadian and metabolic systems.

We show that circadian gene expression of orexin is altered in Firoc mice. This agrees with previous observations that the amount of orexin, but not the number of orexin neurons, is reduced in Firoc mice [29]. In addition, administration of IGF-I at the end of the resting phase reduces the expression of orexin at the beginning of the active phase, indicating that the circadian activity of orexin neurons is influenced by IGF-I levels [43].

Significantly, male Firoc mice showed altered glucose rhythmicity. Similar results were reported in male orexin knockout (KO) mice [44]. Daily blood glucose fluctuations are regulated by the SCN through its glutamatergic and GABAergic projections to the PVN [35,45] and the LH [25], especially to orexin neurons, and their respective para- and sympathetic innervations of the liver. This central regulation promotes a peak in blood glucose, synchronized with the beginning of the active phase [45,46]. We also show that central administration of orexin promotes glucose release, with a more pronounced effect in males. Other authors also reported that activation of orexin neurons in male mice promotes glucose release [26]. Thus, sex-dependent glucose responses to orexin introduce a possible role for estrogen on orexin function, as previously discussed by others [47,48].

Orexin neurons monitor peripheral indicators of energetic status, such as glucose, leptin, or ghrelin serum levels, and modify their activity accordingly, regulating arousal and food seeking behaviors [49]. Here, we show that Firoc mice have altered feeding behavior. Under regular light and food conditions, mild but significant changes were observed in males, reflected in modest changes in the circadian period. The biological relevance of these changes is difficult to evaluate, probably because of the masking effect of light entrainment. Indeed, when the light–dark cycle is uncoupled from the food clock by restricting food availability to the resting phase under constant darkness, feeding rhythms originated by the liver and/or food clock regions [50] synchronize the activity of the animal with the period of food availability. Here, we show that if food is restricted for a 12 h period during the resting phase, a paradigm with mild food clock entrainment [50], Firoc mice showed alterations in the bimodal feeding pattern (regulated by different hypothalamic regions including the LH [51]), which suggests that orexin neurons can impact this behavior. Previous studies have shown that this mild feeding entrainment modifies orexin, MCH, and NPY circadian rhythms [52], and the activity of the respective hypothalamic nuclei [53], reinforcing the notion of a role of IGF-I signaling onto orexin neurons for feeding entrainment. We also showed that Firoc mice under 6 h restricted feeding during the resting phase, a strong food clock entrainment, showed enhanced food anticipatory activity. These results agree with previous ones from our laboratory, showing increased orexinergic activity in Firoc mice undergoing cued behavior paradigms [40]. The FAA phenomenon is independent of the SCN [54,55,56], and only lesions in the DMH abolished it [55]. Hence, FAA requires a network of interacting hypothalamic regions integrating information coming from the periphery. Our results indicate that IGF-I is one of these peripheral signals that orexin neurons sense and integrate into the FAA circuit.

Altered entrainment of circadian and metabolic cycles observed in young Firoc mice preceded in time for the development of metabolic alterations at later times. These include glucose intolerance, hyperglycemia, and altered daily glucose rhythmicity in males, while adult females appear to develop altered lipid metabolism as they become overweight. A sexually dimorphic coupling of the metabolic and circadian cycles has been reported [30], and sexual dimorphism in metabolic [57,58] and circadian activity [59] has also been found. Collectively, these results point to the relevance of IGF-I as a peripheral signal in central glucose homeostasis in conjunction with circadian rhythms.

To start unveiling the mechanisms underlying the role of IGF-I and orexin in coupling the circadian and metabolic cycles, we focused on the family of Krüpel-like transcription factors (KLFs), especially those related to glucose metabolism. The recently described KLF10, KLF15, and KLF11 orchestrate systemic glucose metabolism through local and multi-organ transcriptional regulation [60]. Both KLF10 and KLF15 are known as clock-controlled genes (CCG), which link the clock gene (CG) with metabolism. Further, the expression of KLFs follows a circadian pattern regulated by clock genes and the light cycle [61]. Both KLF transcription factors regulate hepatic glucose production by up- or down-regulating the expression of gluconeogenic enzymes [61]. Central IGF-I administration at the end of the resting phase-modulated liver KLF10 transcription in the control mice, but not in Firoc mice. In contrast, a significant increase in liver KLF15 mRNA was observed only in Firoc mice after IGF-I administration, suggesting that IGF-I acts at a new site in the absence of IGF-I receptors in orexin neurons. Consequently, IGF-I acts at orexin neurons and other as yet unidentified sites to regulate liver KLFs. Although more work is needed, our results suggest that IGF-I influences orexin signaling to synchronize the circadian clock with hepatic metabolism by regulating liver KLF10 expression at the beginning of the active phase [61]. Interestingly, KLF10 KO mice develop alterations in glucose metabolism in a sex-dependent manner, with hyperglycemia in males and obesity in females [61], reassembling the metabolic disorders observed in Firoc mice. We could not determine whether central administration of IGF-I modifies gluconeogenic enzymes in the liver, probably because sampling times need to be accommodated to specifically detect possible changes on these enzymes.

Overall, we observed a new aspect of orexin activity in relation to circadian and metabolic alignment where IGF-I is involved to integrate circadian inputs with metabolic inputs coming from the periphery, mainly the liver, as well as other brain areas, mainly the hypothalamus. Hence, altered orexin activity could be related to IGF-I dysregulation due to aging, diet, or pathology. These results indicate that IGF-I regulates orexin neurons, and, consequently, circadian rhythms and metabolism, extending and confirming previous observations on central actions of IGF-I on mood, metabolism, and wellness [29,40].

## 4. Materials and Methods

### 4.1. Animals

Cre/Lox mice lacking functional IGF-I receptors in orexin neurons (igfrf/f/Orexin Cre: Firoc mice) were obtained, as described in detail elsewhere [27], by crossing Orexin-Cre mice (a kind gift of T. Sakurai, Tsukuba University, Tsukuba, Japan) with IGF-IRf/f mice (B6, 129 background; Jackson Labs; stock number: 012251; exon 3 floxed). Littermates, comprising igfr^f/f^/Orexin-Cre-, igfr^wt/wt^/Orexin-CRE+, and igfir^wt/wt^/Orexine-Cre-, were used as controls. Both males and females were included in the study, as described in each experiment. The estrous cycle of female mice was not controlled. Experiments were conducted during the light phase, except when indicated. Animals were housed separately by sex in standard cages (48 × 26 cm^2^, 5 per cage), and kept in a room with controlled temperature (22 °C) under a 12–12 h light–dark cycle. Mice were fed with a pellet rodent diet and water ad libitum. Animal procedures followed European guidelines (2010/63, European Council Directives) and were approved by the local Bioethics Committee (Government of the Community of Madrid). Animals were randomly allocated in each experiment.

### 4.2. Metabolic Cages

Firoc (3–4 months; *n* = 6–8, both sexes) and control littermates (3–4 months; *n* = 6–7, both sexes) were housed in a comprehensive laboratory animal monitoring system (PhenoMaster, TSE Systems, Bad Homburg, Germany). Locomotor activity, food intake and drinking, energy expenditure (EE), respiratory exchange ratio (RER), and consumed volume of O2 (VO2) were registered. Data were collected over 24 h at 1 h intervals for five days after 1 week of habituation. Analysis of circadian rhythms was performed using the previously described R-based MetaCycle algorithm [29] to estimate the period, the amplitude, and the phase values for each parameter using the data obtained for the last 3–4 days for each parameter.

### 4.3. Behavioral Circadian Test

Mice were housed in individual cages, under normal light conditions before experiments started and ad libitum feeding. In a first experiment, animals were maintained for 1 week under normal (12:12) light and dark regimen, adding food pellets (5 g) into the cage at ZT0 and ZT12, and food consumption was assessed. Then, the light was switched off at ZT0, and the remaining food pellets were weighed at the end of each period. Finally, 1 week later, food was only supplied from ZT0 to ZT12, i.e., their corresponding inactive phase, and food was weighed at ZT12. Five days later, food was weighed at ZT6 and ZT12. In a second experiment to analyze spontaneous activity, mice were housed individually in cages with a running wheel and an automatic wheel counter. Mice were kept under normal conditions for 1 week and running was scored at ZT3, ZT6, ZT9, and ZT12. At the same time, food consumption was measured as described above. After 1 week, lights were switched off at ZT0, and food was provided only for ZT6 to ZT12. The activity was recorded at ZT3, 6, 9, and 12, and calculated as the percentage of the total daily activity. Food anticipatory activity (FAA) was considered as activity during the time (ZT3 to ZT6) before food was supplied and determined after 5 days of light and feeding conditions.

### 4.4. Glucose and Insulin Tolerance Tests

Blood was collected from the tail and glucose levels were determined using Glucomen aero 2K glucometer (A. Menarini Diagnostics, Florence, Italy). For the glucose tolerance test (GTT), mice were fasted for 6 h before injection of a glucose bolus (2 g/kg) intraperitoneally (IP), and blood glucose levels were measured at 0, 5, 15, 30, 90, and 120 min after injection. For the insulin tolerance test (ITT), mice were fasted for 6 h and then 1 U/kg of human insulin (Actrapid Penfill, Novo Nordisk A/S, Bagsværd, Denmark) was IP-injected. Glucose levels were measured at 15, 30, 90, and 120 min after injection. 

### 4.5. Serum Insulin Determination by ELISA

Serum insulin levels were determined by ELISA using ultra-sensitive mouse insulin ELISA kit (#90080, CrystalChem, Inc., Elk Grove Village, IL, USA) following the manufacturer’s instructions. Blood samples were obtained from the facial vein at ZT5, 8, 11, and 15 of mouse maintained under regular food and light conditions. Samples collected during the dark phase were obtained under a dim red light to prevent the circadian alteration of mice.

### 4.6. Body Fat Analysis

Body fat was analyzed as previously described [30,31]. Briefly, mice were anesthetized with isofluorane (2.5% flow rate) via a nose cone setup. Animals were positioned prone with limbs lateral from the torso for uniform computer tomography (CT) scan. Image acquisitions were performed using an Albira II SPECT/CT (Bruker BioSpin PCI, Draria, Algiria). Acquisitions were performed using 600 projections. The X-ray source was set to a current of 400 μA and voltage of 45 kVp. Images were reconstructed using the filtered back projection (FBP) algorithm via the Albira Suite 5.0 Reconstructor using “Standard” parameters. These combined acquisition and reconstruction settings produce a final image with 125 μm isotropic voxels. Images were segmented in POMD v3.3 software according to tissue density; first for the total volume and then for the fat volume segmentation values. Between −500 to −100 Hounsfield units were considered.

### 4.7. Neuronal Cultures

Brains were dissected from embryonic day 15–16 mice as described [62]. Meninges and blood vessels were removed, and the hypothalamus was dissected. Tissue samples were minced with scissors and dissociated with papain at 37 °C for 2 h in agitation. Neuronal cell cultures were kept in a Neurobasal medium supplemented with B27 (Gibco) and Glutamax (Gibco) in 6-well plates coated with poly-L-lysine. Ten days later, cells were washed with PBS and medium replaced with B27-free Neurobasal; 3 h later, cells were treated with IGF-I (Pre-Protech, Cranbury, NJ, USA) at 1 nM for 15 h. After that, plates were washed with PBS, and Trizol was added for RNA extraction.

### 4.8. Intracerebroventricular (ICV) Injections

Guide cannulas (2.5 mm in length, Bilaney Plastic One, Roanoak, VA, USA) were implanted by stereotactic surgery at coordinates 0.2 mm A/P, 0.9 mm M/L, and 2.5 mm in-depth from the skull. Mice were allowed to recover for 1 week before use. For orexin administration, we followed a previously described protocol [44]. Mice were fasted from ZT3 and administered at ZT6. For IGF-I experiments, mice were fasted for 24 h to stimulate orexin activity, and IGF-I was administered at ZT8, as previously reported [35]. At the indicated times, mice were lightly anesthetized to remove the dummy cannula and place the internal cannula. Once mice were awake in the cage (2–3 min), IGF-I (1 µg) in saline solution or orexin-A (3 nmol) (#1455, Tocris Bioscience, Bio-Techne R&D Systems, Minneapolis, MN, USA) diluted in saline were injected at a rate of 1µL/min. The internal cannula remained placed for 1 extra minute before removal. Mice were kept in the cages for 2 h, sacrificed, and cordially perfused, and hypothalamic and liver samples were collected and stored at −80 °C until use. 

### 4.9. Virus Injection

For inactivation of IGF-IR in orexin neurons of adult mice, we bilaterally injected the lateral hypothalamus (stereotaxic coordinates: AP = −1.4; ML = ±0.9; DV= −5.4) of IGF-IR^f/f^ mice (3 months old), an AAV-HCRT-Cre-EGFP virus (AAV-HCRT-CRE), or an AAV-HCRT-EGFP virus (AAV-control). One month later, the GTT was performed, as described above. AAV-HCRT-CRE-EGFP and AAV-HCRT-EGFP viruses were packaged at the Viral Vector Production Unit, UAB-VHIR (Barcelona, Spain), as previously described [40].

### 4.10. RNA Isolation and Real-Time PCR

Tissue RNA was extracted with Trizol (Life Technologies, Carlsbad, CA, USA), as described elsewhere [63]. cDNA was synthesized from 1 μg of RNA of each sample following the manufacturer’s instructions (High-Capacity cDNA Reverse Transcription Kit; Applied Biosystems, Waltham, MA, USA). Fast real-time qPCR was performed using the SYBR Green method (Fast SYBR Green Master Mix, Applied Biosystems, Waltham, MA, USA) with the QuantStudio 3 Real-Time PCR System (Applied Biosystems, Waltham, MA, USA). Relative mRNA expression was determined by the 2−ΔΔCT method [64] and normalized to ribosomal 18S mRNA levels.

### 4.11. Statistics

Statistical analyses were performed with OriginPro (OriginLab Corp., Northhampton, MA, USA). The F-Test or Levene’s test were used to determine the variance homogeneity before the Student’s *t*-test, which compared two groups using either 1- or 2-way ANOVA (for more than 2 groups) and the Tukey post-hoc (for multiple comparisons). Results are shown as mean ± standard error (SEM) and significant values as: * *p* < 0.05; ** *p* < 0.01, and *** *p* < 0.001.

## Supplementary Materials

The following supporting information can be downloaded at: https://www.mdpi.com/article/10.3390/ijms23094679/s1.
