# Supplementary material for: Insulin-like Growth Factor I Couples Metabolism with Circadian Activity through Hypothalamic Orexin Neurons
Source: Int J Mol Sci. 2022 Apr 23;23(9):4679. doi: 10.3390/ijms23094679 (PMC9101627; doi:10.3390/ijms23094679)
Supplement: Supplementary file 1 [file ijms-23-04679-s001.zip › ijms-1684933-supplementary.pdf]

(a) Males

	Period		Phase		Amplitude	
	Firoc	Control	Firoc	Control	Firoc	Control
Feeding	25.88 ± 0.59	23.55 ± 1.34	14.22 ± 0.64	15.32 ± 0.69	0.44 ± 0.07	0.41 ± 0.06
Water	28.00 ± 0.00	28.00 ± 0.00	24.00 ± 0.00	24.00 ± 0.00	13.94 ± 1.38	16.19 ± 2.61
RER	23.63 ± 0.85	22.33 ± 0.76	15.26 ± 0.74	16.76 ± 0.84	1.13 ± 0.03	1.11 ± 0.05
EE	23.94 ± 0.72	22.72 ± 0.85	15.62 ± 0.27	16.16 ± 0.56	19.92 ± 0.50	19.93 ± 1.02
VO2	23.76 ± 0.70	22.56 ± 0.64	15.71 ± 0.29	15.70 ± 0.36	3878.26 ± 86.64	3844.45 ± 179.52
Activity	23.60 ± 0.94	23.57 ± 1.07	16.89 ± 0.76	16.82 ± 1.23	1903.86 ± 261.95	1928.81 ± 438.47

(b) Females

	Period		Phase		Amplitude	
	Firoc	Control	Firoc	Control	Firoc	Control
Feeding	20.96 ± 0.67	25.40 ± 1.14	15.02 ± 0.87	16.01 ± 1.35	0.48 ± 0.06	1.83 ± 1.41
Water	28.00 ± 0.00	28.00 ± 0.00	23.73 ± 0.27	24.00 ± 0.00	15.23 ± 2.37	15.43 ± 2.51
RER	23.81 ± 1.54	22.59 ± 0.92	16.56 ± 1.64	14.96 ± 0.53	1.15 ± 0.04	1.14 ± 0.03
EE	23.76 ± 0.65	23.24 ± 0.69	15.60 ± 0.50	16.07 ± 0.57	21.88 ± 1.75	24.43 ± 0.96
VO2	23.65 ± 0.69	24.96 ± 0.99	15.54 ± 0.47	18.41 ± 1.50	4239.86 ± 321.69	4684.42 ± 139.36
Activity	21.92 ± 0.68	24.54 ± 1.06	15.55 ± 0.40	18.21 ± 1.50	3045.31 ± 782.06	3199.75 ± 483.77

Table S1: * **Algorithmic analysis of metabolic circadian rhythms.** Data obtained from Phenomaster metabolic cages from male (n=8 per genotype group) and female (n=8 per genotype group) Firoc and control mice were analyzed using Metacycle algorithm. Estimated Mean and SEM of the period, phase, and amplitude parameters from feeding, water intake, RER, EE, activity, and oxygen volume consumed (VO2) are shown.

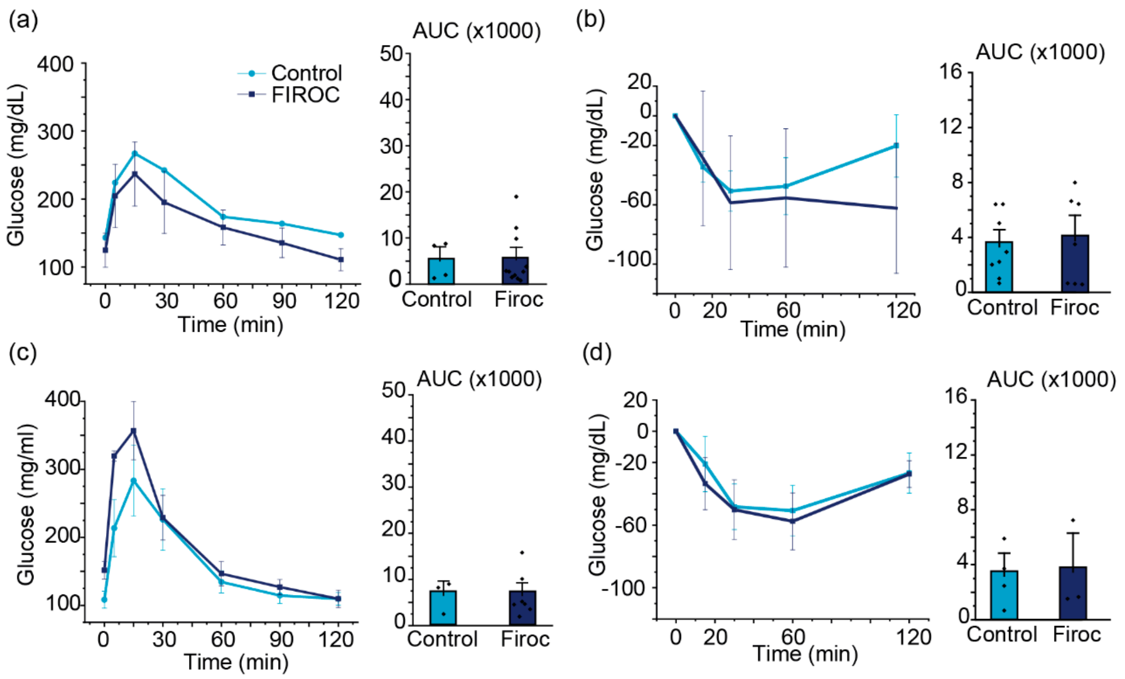

Figure S1: Glucose metabolism is preserved in young Firoc mice. (a) Glucose tolerance test in young (< 6 months) male mice. Blood glucose levels and quantification histograms (area under the curve: AUC) are shown. **(b)** Insulin tolerance test in young males. Blood glucose levels and quantification histograms (AUC) are shown. **(c)** Glucose tolerance test in young females. Blood glucose levels and quantification histograms are shown. **(b)** Insulin tolerance test in young females. Blood glucose levels and quantification histograms are shown. Neither young Firoc males (a) nor females (c), showed altered glucose responses throughout the 2 h test. Glucose levels after systemic insulin administration remained within normal values in both young male (b) and young female (d) Firoc mice.

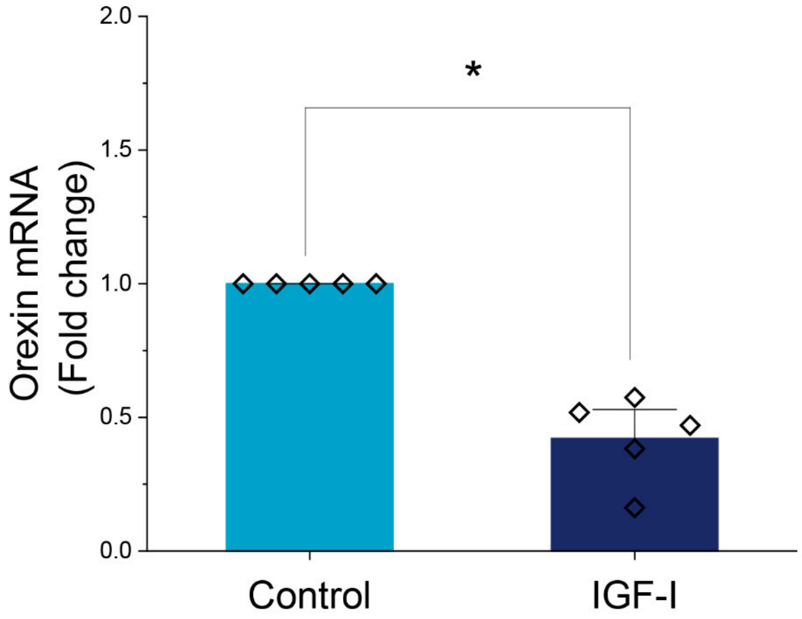

Figure S2: IGF-I inhibits orexin expression in hypothalamic neurons. Hypothalamic neurons from E13 mice embryos (n=5) were cultured for 10 days. Cultures were then treated with IGF-I (1 nM) or saline, and orexin mRNA levels were measured 15 h later (mean ± SEM, two-sample t-test, *p< 0.05).

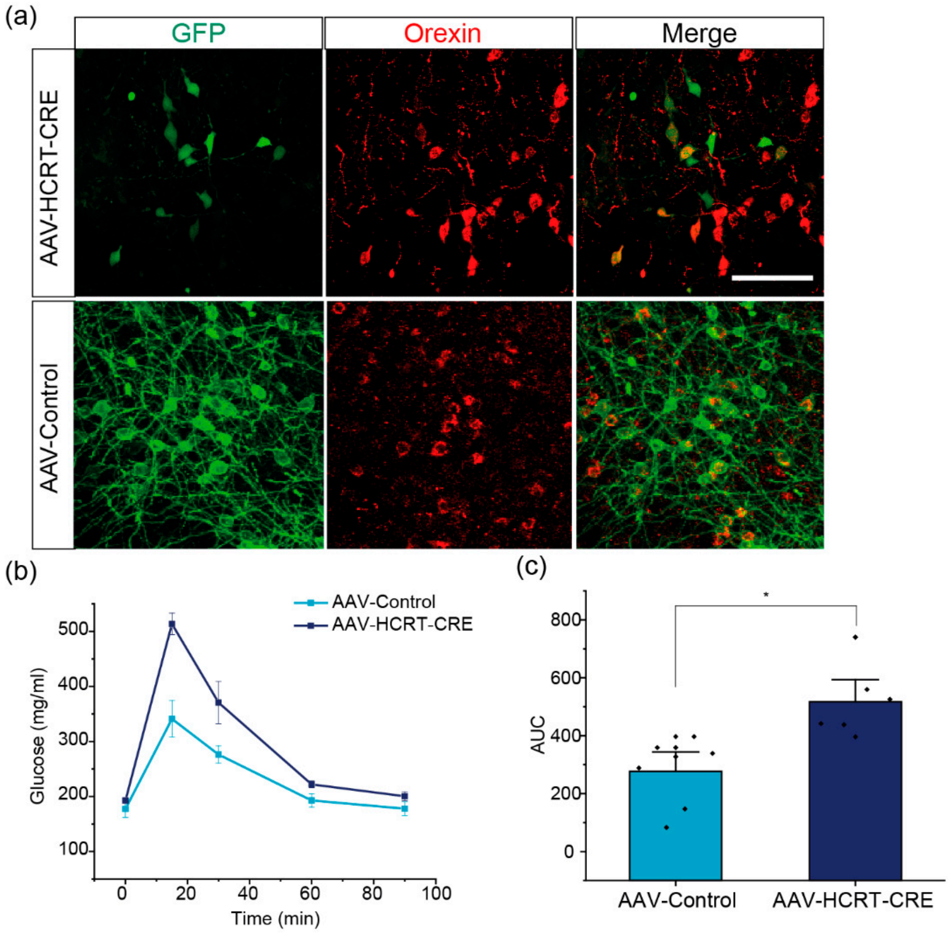

Figure S3: Igfir$^{f/f}$ mice transduced with AAV-HCRT-CRE viral vector also display glucose intolerance. Bilaterally injected igfir$^{f/f}$ mice with AAV-HCRT-CRE (n=6) or AAV-Control virus (n=7) **(a)** Immunohistochemistry showing specific expression of green fluorescent protein (Green) in orexin neurons (red) after AAV-HCRT-CRE injection (upper panel) or AAV-Control virus (bottom panel). **(b)** Transduced mice were assayed for glucose tolerance test. Glucose bolus (2g/Kg) was administered after 6h fasting and blood glucose levels were measured from the tail at 15, 30, 60, and 90 min after administration. **(C)** mice injected with AAV-HCRT-CRE virus showed significant glucose intolerance with respect to the AAV-Control injected mice (mean AUC ± SEM, two-sample t-test, *$p < 0.05$).
